# Characteristics and Outcomes of IgA Nephropathy Patients With SGLT2 Inhibitor-Induced Polycythemia

**DOI:** 10.7759/cureus.91789

**Published:** 2025-09-07

**Authors:** Yoshitaka Furuto, Akio Namikawa, Dai Sato, Yuko Shibuya

**Affiliations:** 1 Department of Hypertension and Nephrology, NTT Medical Center Tokyo, Tokyo, JPN

**Keywords:** blood pressure, cardiovascular disease (cvd), chronic kidney disease, hematopoietic effect, iga nephropathy, polycythemia, proteinuria, sglt2 inhibitor

## Abstract

Purpose: Sodium-glucose co-transporter 2 (SGLT2) inhibitors provide renal and cardiovascular benefits in IgA nephropathy (IgAN). However, their hematopoietic effect can cause polycythemia in rare cases. We aimed to evaluate the clinical characteristics and two-year outcomes of patients with IgAN with SGLT2 inhibitor-induced polycythemia.

Methods: We retrospectively analyzed data of adult patients with IgAN treated with dapagliflozin (DAPA) at our department between 2021 and 2023. Patients were divided into two groups based on whether or not they developed polycythemia within 24 months of initiating DAPA therapy (those who developed polycythemia were in the polycythemia (P) group, and those who did not were in the normal (N) group). Clinical characteristics and cardio-renal outcomes were evaluated at baseline and three, six, 12, 18, and 24 months post-SGLT2 inhibitor initiation and compared between the groups.

Results: Of the 174 patients screened, 28 were included. Compared to the N group (n=18), the P group (n=10) had a higher proportion of men, dyslipidemia, and hyperuricemia, and higher body weight and higher red blood cell count at baseline. Polycythemia developed even in patients with high baseline hemoglobin (Hb) levels; however, these returned to baseline levels within two years. Systolic blood pressure and proteinuria levels decreased significantly over the two-year period in both groups, but the decrease was slower in the P group than in the N group. No cardiovascular events occurred in either group during the follow-up period.

Conclusions: The SGLT2 inhibitor-induced polycythemia in patients with IgAN was transient and did not affect the two-year cardio-renal outcomes. Therefore, our findings suggest that continuing SGLT2 inhibitor therapy in these patients may be reasonable, as the condition appeared to be transient and did not adversely affect cardio-renal outcomes.

## Introduction

Sodium-glucose co-transporter 2 (SGLT2) inhibitors, in combination with renin-angiotensin system (RAS) inhibitors, are established as first-line therapy for patients with chronic kidney disease (CKD) and have demonstrated efficacy in those with IgA nephropathy (IgAN) [1,2]. These inhibitors exert renal protective effects in CKD by ameliorating glomerular hyperfiltration through enhanced tubuloglomerular feedback, upregulating sirtuin 1 (SIRT1) and 5'-adenosine monophosphate-activated protein kinase (AMPK) signaling pathways, and improving anemia by increasing erythropoietin (EPO) production, enhancing iron utilization, and suppressing hepcidin activity [3,4]. The hematopoietic effects of SGLT2 inhibitors serve as a surrogate marker of renal protection [5] and play a critical role in improving outcomes in heart failure [6]. Anemia is a common complication in advanced CKD due to reduced red blood cell (RBC) production, impaired RBC metabolism, decreased EPO synthesis, EPO resistance, and iron deficiency [7]. Effective management of renal anemia not only slows the progression of renal function decline but also reduces cardiovascular disease (CVD) events and mortality [8]. Notably, SGLT2 inhibitors have shown promise in managing renal anemia in advanced CKD, potentially delaying the need for iron supplementation and erythropoiesis-stimulating agents (ESAs) [9,10]. Furthermore, in patients without renal anemia, SGLT2 inhibitor administration increases hemoglobin (Hb) and hematocrit (Ht) levels while conferring protective effects on the heart and kidneys and reducing the incidence of CVD events [3].

Although patients with CKD without anemia may develop polycythemia during SGLT2 inhibitor therapy in rare cases, it is generally recommended to continue treatment unless the patient has polycythemia vera [11,12]. In contrast, excessive Hb level elevation due to ESAs or hypoxia-inducible factor (HIF)-prolyl hydroxylase inhibitors (PHIs) in patients with CKD is associated with an increased risk of CVD events and should be avoided [13-19]. This difference may reflect a different hematopoietic mechanism or fluid environment of SGLT2 inhibitors compared with ESAs or HIF-PHIs. In addition, the NephroTest study [20] reported that polycythemia was observed in 3.5% of patients with IgAN, a disease associated with polycythemia [21]. This relationship may result from the interaction between highly polymerized galactose-deficient IgA1 and transferrin receptor 1, which increases the sensitivity of erythroid precursor cells to EPO [22].

The effects of SGLT2 inhibitors on hematopoiesis in patients with IgAN are not fully understood, rendering uncertainties in the clinical management of polycythemia during SGLT2 inhibitor therapy, particularly in terms of treatment continuation. Therefore, this study aimed to detail the clinical characteristics and outcomes of patients with IgAN with SGLT2 inhibitor-induced polycythemia in order to evaluate the benefits of continuing SGLT2 inhibitor treatment.

## Materials and methods

Study design and population

This is a retrospective, single-center study conducted at NTT Medical Center Tokyo, Japan. The study protocol was approved by the Ethical Review Board of NTT Medical Center Tokyo (approval no. 000200019199-01). Owing to the retrospective study design, the requirement for formal consent was waived; however, patients were given the option to opt out by indicating their consent to participate via the hospital’s website.

We retrospectively reviewed the electronic medical records of adult patients with IgAN treated at the Department of Nephrology at NTT Medical Center Tokyo between 2021 and 2023. The diagnosis of IgAN was confirmed by nephrologists and pathologists based on renal biopsy findings. Patients who consecutively received SGLT2 inhibitor treatment for IgAN for at least two years were included. The exclusion criteria were as follows: 1) use of steroids or immunosuppressive agents; 2) advanced CKD (G4 or higher); 3) severe proteinuria (urinary protein ≥ 3 g/gCr); 4) age 70 or older; 5) presence of malignancies, chronic infections, polycythemia vera, or bleeding disorders; 6) use of iron preparations or hematopoietic agents such as ESAs or HIF-PHIs; 7) poorly controlled hypertension (≥ 150/100 mmHg); 8) follow-up period less than 24 months or incomplete medical records; and 9) changes in medications affecting body weight (BW), blood pressure (BP), blood concentrations, renal function, or proteinuria during the study period.

Data collection and definitions

Demographic data, including age, sex, BW, and smoking status, and clinical data, including BP measurements, medical history (CVD events, hypertension, diabetes, hyperlipidemia, and hyperuricemia), concomitant medications, blood test results, and urinalysis findings, were extracted from the medical records. Polycythemia was defined as an Hb level > 16.5 g/dL in men and 16.0 g/dL in women. The cohort was divided into two groups. Patients who developed polycythemia within 24 months of initiating SGLT2 inhibitor treatment were in the polycythemia (P) group, and those who did not develop it were in the normal (N) group.

Physicians and hospital nurses provided standardized instructions for BP measurement. Validated automated oscillometric upper-arm devices were recommended for accuracy. Patients were instructed to record their BP after at least two minutes of rest in the morning, within one hour of waking. All parameters, including BW, BP, Hb, and Ht levels, estimated glomerular filtration rate (eGFR), proteinuria, and incidence of CVD events (e.g., heart disease, stroke, and cardiac death), were assessed at baseline and at three, six, 12, 18, and 24 months following SGLT2 inhibitor therapy initiation.

Statistical analysis

Data were summarized as mean ± standard deviation for continuous variables and frequencies with percentages for categorical variables. Continuous variables were analyzed using paired Student’s t-tests or one-way analysis of variance, as appropriate, while categorical variables were compared using Pearson’s chi-squared test. Data were rounded to two decimal places. Statistical analyses were performed using Microsoft Excel (Microsoft Corp., Redmond, WA, USA), with significance set at p < 0.05. For categorical variables, Pearson’s chi-squared test was used, and results are reported with chi-squared values, degrees of freedom, p-values, and effect sizes (φ for 2×2 tables). When expected counts were <5, Fisher’s exact test was preferred, and odds ratios (OR) with 95% confidence intervals were provided.

## Results

Patient characteristics

Among the 174 patients with IgAN treated during the study period, 92 consecutively received dapagliflozin (DAPA) at a dose of 10 mg for IgAN. Among them, after excluding patients aged 70 or older (n=3), those who used steroids or immunosuppressive agents (n=30), those with advanced CKD (n=4), severe proteinuria (n=2), malignancy (n=1), poorly controlled hypertension (n=2), and changes of medications (n=5), as well as patients with a follow-up duration less than 24 months or incomplete medical records (n=19), 28 patients were included in the analysis. Baseline characteristics are summarized in Table 1, where continuous variables are shown as mean ± SD with p-values, and categorical variables are presented with Fisher’s exact test results and odds ratios (95% CI). Corresponding chi-square statistics with degrees of freedom and effect sizes (φ) are described in the text.

**Table 1:** Baseline clinical characteristics of patients with IgAN IgAN: IgA nephropathy; sBP: Systolic blood pressure; dBP: Diastolic blood pressure; HR: Heart rate; BW: Body weight; BMI: Body mass index; Alb: Albumin; UA: Uric acid; eGFR: Estimated glomerular filtration rate; TC: Total cholesterol; HbA1c: Glycated hemoglobin (HbA1c); RBC: Red blood cell; Hb: Hemoglobin; Ht: Hematocrit; Plt: Platelets; Na: Sodium; K: Potassium; Cl: Chloride; Ca: Calcium; IP: Inorganic phosphorus Notes: Continuous variables were analyzed using t-tests or ANOVA. Categorical variables were analyzed with Fisher’s exact test (preferred when expected counts <5). Odds ratios (OR) with 95% confidence intervals are provided for 2×2 comparisons. For variables with significant between-group differences, chi-square statistics with degrees of freedom and effect sizes (φ) are additionally reported in the main text.

Characteristics	Normal (N) group (n=18)	Polycythemia (P) group (n=10)	p-value / Fisher p	OR (95% CI)
Age (years)	50.7 ± 11.4	52.8 ± 7.1	0.61	—
sBP (mmHg)	129.6 ± 13.6	128.2 ± 8.4	0.78	—
dBP (mmHg)	83.4 ± 13.1	80.2 ± 6.8	0.49	—
HR (beats/min)	70.7 ± 8.2	78.4 ± 11.2	0.054	—
BW (kg)	63.1 ± 9.1	70.6 ± 7.8	0.042	—
BMI (kg/m2)	23.1 ± 1.7	24.7 ± 2.2	0.054	—
Alb (g/dL)	4.12 ± 0.19	4.26 ± 0.22	0.11	—
UA (mg/dL)	5.8 ± 0.5	6.6 ± 0.7	0.003	—
eGFR (ml/min/1.73m2)	52.8 ± 12.2	49.4 ± 13.3	0.52	—
TC (mg/dL)	195.1 ± 20.8	174.0 ± 15.8	0.012	—
HbA1c (%)	5.5 ± 0.3	5.5 ± 0.2	0.58	—
RBC (×10^4/μL)	445.3 ± 41.5	527.8 ± 27.5	<0.0001	—
Hb (g/dL)	13.6 ± 0.9	16.4 ± 0.8	<0.0001	—
Ht (%)	41.1 ± 2.7	49.2 ± 2.6	<0.0001	—
Plt (×10^4/μL)	24.7 ± 4.4	25.0 ± 2.6	0.88	—
Na (mmol/L)	141.0 ± 1.7	139.8 ± 0.7	0.11	—
K (mmol/L)	4.39 ± 0.32	4.62 ± 0.20	0.051	—
Cl (mmol/L)	105.1 ± 1.9	104.0 ± 1.4	0.13	—
Ca (mg/dL)	9.4 ± 0.29	9.4 ± 0.29	0.74	—
IP (mg/dL)	3.18 ± 0.55	3.5 ± 0.46	0.14	—
Proteinuria (g/gCr)	0.87 ± 0.65	0.85 ± 0.60	0.93	—
Male, n (%)	8 (44.4)	10 (100)	0.0039	0.04 (0.00–0.76)
Smoking, n (%)	6 (33.3)	2 (20.0)	0.6692	2.00 (0.32–12.51)
Hypertension, n (%)	10 (55.6)	6 (60.0)	1.0000	0.83 (0.17–4.01)
Hyperlipidemia, n (%)	10 (55.6)	10 (100)	0.0251	0.06 (0.00–1.16)
Hyperuricemia, n (%)	12 (66.7)	6 (60.0)	1.0000	1.33 (0.27–6.61)
Calcium channel blocker, n (%)	4 (22.2)	6 (60.0)	0.0974	0.19 (0.03–1.25)
β blocker, n (%)	2 (11.1)	0 (0)	0.5238	3.07 (0.13–72.34)
Statin, n (%)	10 (55.6)	10 (100)	0.0251	0.06 (0.00–1.16)
Antihyperuricemics, n (%)	12 (66.7)	6 (60.0)	1.0000	1.33 (0.27–6.61)

The mean age of the total cohort was 51.4±10.1 years, and 64.2% were male. None of the patients had a history of CVD, and all were receiving RAS inhibitors at baseline, with a mean BP of 129.1±12/82.3±11.4 mmHg. Baseline hematological and renal function parameters included a mean RBC count of 474.8±54.2 × 104/µL, an Hb level of 14.6±1.6 g/dL, an Ht of 44.0±4.7%, an eGFR of 51.6±12.7 ml/min/1.73 m², and proteinuria of 0.87±0.63 g/gCr.

Among the 28 patients included, 10 (35.7%) did (P group) and 18 (64.3%) did not develop (N group) polycythemia within 24 months of DAPA therapy initiation. None of the patients exhibited symptoms or clinical features suggestive of polycythemia vera, such as leukocytosis, thrombocytosis, pruritus, or splenomegaly. Compared to the N group, the P group exhibited a higher proportion of males, higher BW, higher prevalence of hyperlipidemia, hyperuricemia, and statin use, and higher baseline RBC, Hb, and Ht levels. Baseline BP, eGFR, and proteinuria levels were similar in the two groups. Sleep apnea syndrome was not assessed, and smoking rates did not differ between the groups. No other significant differences in baseline characteristics were observed. The proportion of male patients was significantly higher in the polycythemia group compared with the normal group (χ²(1, N = 28) = 8.64, p = 0.003, φ = 0.56). Similarly, the prevalence of hyperlipidemia (χ²(1, N = 28) = 6.22, p = 0.013, φ = 0.47) and statin use (χ²(1, N = 28) = 6.22, p = 0.013, φ = 0.47) were significantly higher in the polycythemia group. No significant between-group differences were observed for smoking status, hypertension, hyperuricemia, calcium channel blocker or β-blocker use, or antihyperuricemic agent use (all p > 0.05). Corresponding chi-square statistics with 1 degree of freedom were low (χ² values ranging from 0.00 to 1.10), with small effect sizes (φ < 0.20), indicating negligible between-group differences.

Patient outcomes according to polycythemia development

**Figure 1:**
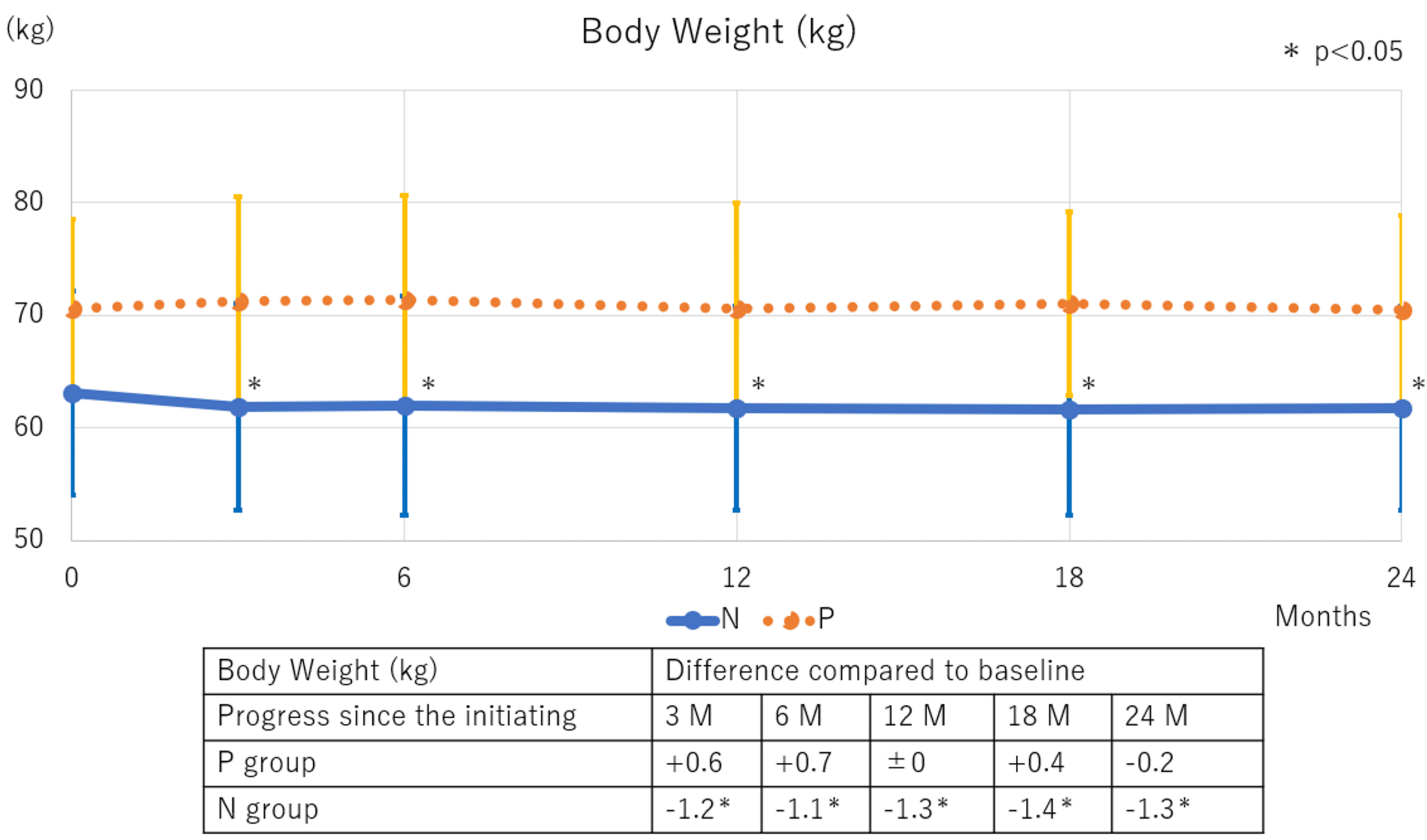
Changes in BW following SGLT2 inhibitor therapy initiation The N group exhibited a significant weight loss at three months compared to baseline, while the P group showed no change in BW. BW: Body weight; SGLT2: Sodium-glucose co-transporter 2; M: Months; N: Normal; P: Polycythemia

**Figure 2:**
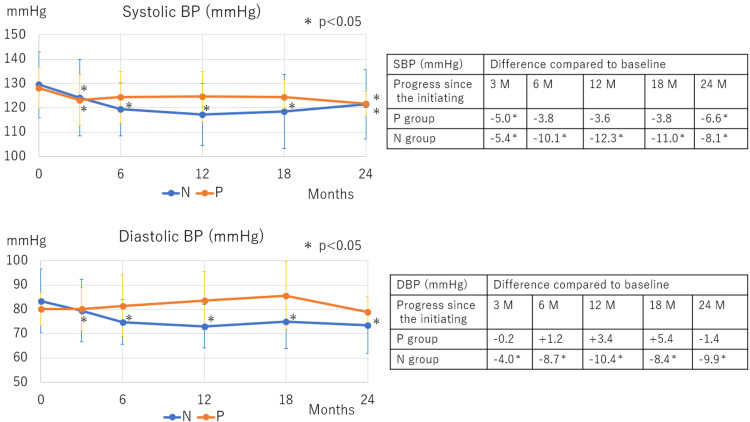
Changes in BP following SGLT2 inhibitor therapy initiation In the N group, systolic BP significantly decreased from three months onwards. In the P group, a significant decrease was observed only at three and 24 months. Diastolic BP decreased significantly from three months onward in the N group, but no change was observed in the P group. M: Months; N: Normal; P: Polycythemia; DBP: Diastolic blood pressure; SBP: Systolic blood pressure

Over 24 months of DAPA treatment, compared with baseline values, significant BW reduction was observed in the N group starting at three months, whereas no BW loss occurred in the P group (Figure 1). Regarding BP changes compared with baseline values, systolic BP reduction was noted in both groups, with the significant decrease starting at three months and 24 months after DAPA therapy initiation in the N and P groups, respectively. Diastolic BP significantly decreased in the N group starting at three months but remained unchanged in the P group (Figure 2).

**Figure 3:**
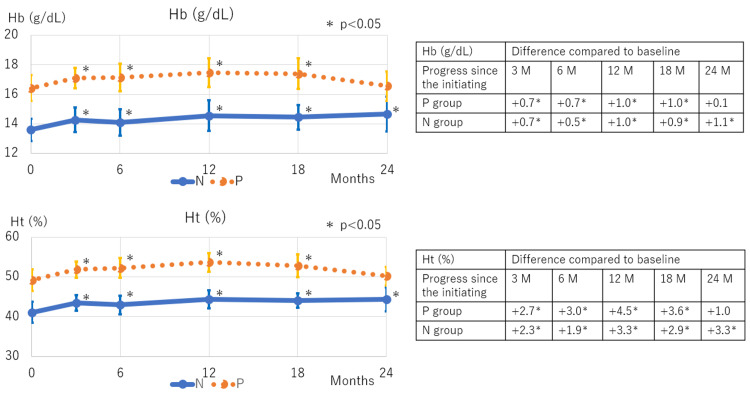
Trends in Hb and Ht levels following SGLT2 inhibitor therapy initiation The N group showed a significant increase in Hb and Ht levels from three months onward. In the P group, a significant increase was observed from three to 18 months, but at 24 months, there was no significant difference from baseline. SGLT2: Sodium-glucose co-transporter 2; M: Months; N: Normal; P: Polycythemia; Hb: Hemoglobin; Ht: Hematocrit

**Figure 4:**
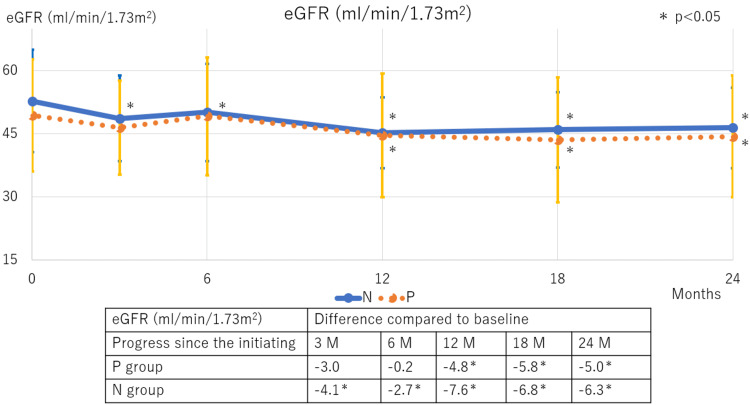
Trends in eGFR following SGLT2 inhibitor therapy initiation The N group showed a significant decrease in eGFR after three months compared to baseline. In the P group, a significant decrease was observed after 12 months. eGFR: Estimated glomerular filtration rate; SGLT2: Sodium-glucose co-transporter 2; M: Months; N: Normal; P: Polycythemia

**Figure 5:**
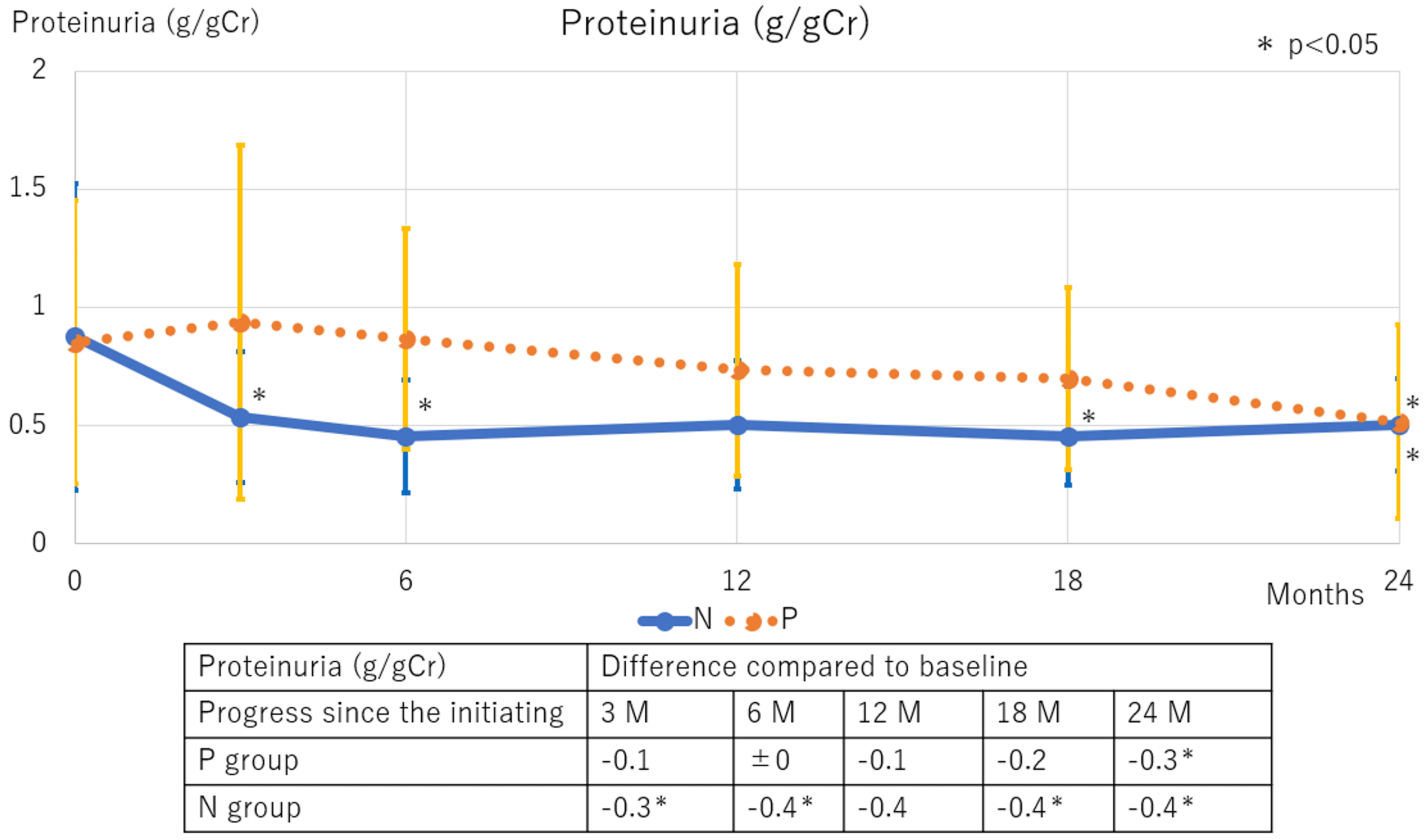
Changes in proteinuria levels following SGLT2 inhibitor therapy initiation The N group showed a significant decrease in proteinuria levels from three months onward. In the P group, no significant decrease was observed from three to 18 months. However, a significant decrease was noted at 24 months. *Indicates a significance level of p < 0.05. SGLT2: Sodium-glucose co-transporter 2; M: Months; N: Normal; P: Polycythemia

Among hematological parameters, Hb and Ht levels increased in both groups following DAPA therapy initiation, with similar magnitudes of change observed up to 18 months. At 24 months, the polycythemia resolved in the P group, with Hb and Ht levels returning to baseline values (Figure 3). In terms of renal function, the eGFR significantly decreased in the N group at three months and in the P group between 12 and 24 months (Figure 4). Proteinuria levels significantly declined in both groups, with a consistent decline in the N group from three months onwards, and a gradual, slower decline in the P group (Figure 5).

## Discussion

Our findings revealed that patients with IgAN who developed polycythemia following SGLT2 inhibitor therapy initiation were predominantly male and had higher BW, dyslipidemia, and hyperuricemia at baseline, suggesting a potential link with hypercaloric intake. None of these patients were diagnosed with polycythemia vera. Regarding outcomes, patients with IgAN and SGLT2 inhibitor-induced polycythemia did not experience significant increases in BP or the incidence of adverse events after DAPA initiation. The polycythemia was transient and resolved within 24 months. Importantly, no CVD events were reported in either group during the 24-month follow-up period.

Despite the transient polycythemia in the P group, significant reductions in systolic BP and proteinuria levels were observed after 24 months of SGLT2 inhibitor therapy, highlighting the continued therapeutic utility of SGLT2 inhibitors in IgAN. Importantly, while the decline in proteinuria levels was initially slower in the P group, both groups exhibited comparable proteinuria levels by 24 months. These findings suggest that SGLT2 inhibitor therapy was effective in both groups, with sustained cardio-renal benefits despite the transient polycythemia in the P group. It emphasizes the benefits of sustained SGLT2 inhibitor use in IgAN patients with SGLT2 inhibitor-associated polycythemia, excluding polycythemia vera [21].

To the best of our knowledge, this study is the first to evaluate the long-term efficacy of SGLT2 inhibitors in IgAN patients with SGLT2 inhibitor-associated polycythemia. Several key findings emerged. First, despite the transient polycythemia, BW reduction was not observed in the P group, and systolic BP declined significantly, suggesting mechanisms of BP reduction beyond natriuresis, glucosuria, or BW reduction, potentially involving improvements in arterial stiffness, endothelial function, and sympathetic nervous system modulation [23]. Second, the polycythemia resolved within 24 months without significant differences in Hb and Ht levels at the end of follow-up compared with baseline. This is consistent with previously reported findings. Namely, SGLT2 inhibitors have been shown to attenuate polycythemia through adipocyte reduction and suppression of HIF-1α expression, mechanisms implicated in obesity-related polycythemia [24-26]. In patients with higher baseline Ht levels, a transient increase was observed following SGLT2 inhibitor initiation, but long-term therapy optimized Ht levels [27]. Third, both groups exhibited similar magnitude increases in Hb and Ht levels from three to 18 months post-SGLT2 inhibitor initiation. Interestingly, while the P group demonstrated higher Hb and Ht levels, the Hb increase was not significantly different from that in the N group. Previous studies have consistently reported that Hb levels increase with the use of SGLT2 inhibitors regardless of the presence or absence of anemia [28-31], and this was also the case in IgAN. The SGLT2 inhibitors elevate Hb and Ht levels across diverse patient populations, including those with type 2 diabetes, CKD, and heart failure, irrespective of eGFR [9,27]. We speculate that this hematopoietic effect may involve HIF-2α upregulation via SIRT1 activation, while adipocyte reduction and HIF-1α suppression contribute to polycythemia resolution.

The mechanisms underlying SGLT2 inhibitor-induced hematopoiesis differ from those of ESAs and HIF-PHIs, particularly in their reliance on SIRT1 and AMPK. The SGLT2 inhibitors enhance erythropoiesis via three primary pathways: endogenous EPO production, improved iron utilization, and hepcidin inhibition. Endogenous EPO synthesis is driven by renal cortical reoxygenation and transcriptional activation of the EPO gene through HIF-2α upregulation mediated by SIRT1 signaling [32]. Experimental and clinical studies of CKD associate decreased HIF-2α expression and elevated HIF-1α expression with renal injury, inflammation, and cardiovascular dysfunction [33-35]. By activating SIRT1 and AMPK signaling, SGLT2 inhibitors reduce oxidative stress, inflammation, and fibrosis and improve mitochondrial function and autophagy, mitigating CKD progression and CVD risk [36-38]. Consequently, upregulation of HIF-1α may be unnecessary [39]. Iron sufficiency remains essential for maintaining RBC quality, although recent findings indicate that SGLT2 inhibitor-induced erythropoiesis persists despite iron deficiency, potentially through enhanced cytoplasmic iron utilization (ferritinophagy) via increased autophagic flux mediated by SIRT1 signaling [40,41].

In a large-scale study, SGLT2 inhibitor therapy significantly increased polycythemia prevalence without elevating CVD risk [31]. In patients with obstructive sleep apnea, who have high EPO concentrations and a high risk of polycythemia [42], administration of SGLT2 inhibitors reduced the risk of CVD events despite the increased Ht levels seen in some patients. However, no data have been reported on the changes in Ht levels after administration of SGLT2 inhibitors in this patient population [43]. A study of 100 patients with SGLT2 inhibitor-induced polycythemia reported 10 thrombotic events over a two-year follow-up period, independent of Hb levels [12], suggesting that discontinuation due to polycythemia may not be warranted [11,12].

Unlike traditional polycythemia, SGLT2 inhibitor-associated polycythemia does not heighten CVD risk, likely due to cardioprotective effects, reduced oxidative stress, and modulation of inflammatory pathways [8]. Furthermore, changes in RBC rheology may underpin these benefits. The SGLT2 inhibitors reduce interstitial fluid excess while maintaining intravascular volume, distinguishing their effects from those of traditional diuretics and mitigating the risk of CVD, even in the presence of polycythemia [44]. Blood viscosity is influenced by plasma viscosity, Ht, and red blood cell deformability (RBCD). The latter, determined by the surface area-to-volume ratio, internal viscosity, and erythrocyte membrane fluctuation, plays a pivotal role in RBC passage through microcirculation [45]. Young RBCs, characterized by enhanced RBCD and optimal geometry, improve oxygen delivery and axial flow, while mature RBCs with reduced deformability and diminished surface-area-to-volume ratios are prone to wall collisions and inefficient oxygen transport, predisposing them to thrombus formation [46]. While polycythemia typically increases blood viscosity and may compromise oxygen transport efficiency when Ht exceeds 38% [47], RBCD deficits in conditions such as diabetes, nephrotic syndrome, and CKD exacerbate viscosity and elevate CVD risk [10,48]. Emerging evidence suggests that SGLT2 inhibitors improve RBCD by enhancing erythrocyte membrane fluctuation, thereby mitigating viscosity even in polycythemia [49]. Additionally, SGLT2 inhibitors ameliorate endothelial dysfunction and inhibit platelet activation [50]. The RBC aggregation, driven by reduced RBCD, oxidative stress, inflammation, or proteolytic enzyme-mediated alterations in RBC surface properties [45], may also be counteracted by SGLT2 inhibitors through their humoral and rheological benefits, potentially preventing CVD risk escalation. Further research is warranted to elucidate the mechanism underlying RBCD improvements with SGLT2 inhibitor therapy.

In this study, polycythemia was defined as an Hb level greater than 16.5 g/dL in men and 16.0 g/dL in women. In our cohort, polycythemia was observed only in men. While polycythemia in general is associated with an increased risk of cardiovascular and thrombotic events, SGLT2 inhibitor-induced polycythemia may differ mechanistically from secondary polycythemia or polycythemia vera. Our findings, consistent with prior reports, suggest that this condition is usually transient (see Appendix A) and not associated with adverse cardiovascular outcomes.

This study has several limitations. First, the sample size was relatively small, and the study population was biased toward certain patient characteristics, which may limit the generalizability of our findings. Second, as a single-center retrospective study, the potential influence of bias and unmeasured confounding factors cannot be fully excluded. Therefore, our results should be interpreted with caution and confirmed in larger, prospective, multicenter studies. Third, histological evaluation of IgAN severity by renal biopsy was not included because prior biopsy data were lacking and assessment methods were heterogeneous. In addition, patients with advanced CKD (stage G4 or higher) were excluded, as the prevalence of polycythemia is generally low in this population. Furthermore, the presence of JAK2 mutations was not examined because none of the patients exhibited clinical features consistent with polycythemia vera. Finally, we speculate that the proportion of polycythemia in our cohort may have been relatively high because such patients are often followed for longer durations owing to concerns about disease progression.

## Conclusions

Among patients with IgAN, those who developed polycythemia after SGLT2 inhibitor therapy were predominantly male and exhibited high BW, dyslipidemia, hyperuricemia, and high RBC, Hb, and Ht levels at baseline. The SGLT2 inhibitor therapy in this population resulted in transient polycythemia, reduced systolic BP and urinary protein excretion, and no CVD events, suggesting favorable outcomes. Therefore, our findings suggest that continuing SGLT2 inhibitor administration in patients with IgAN who develop SGLT2 inhibitor-induced polycythemia may be reasonable, as the condition appeared to be transient and did not adversely affect cardio-renal outcomes.

## Figures and Tables

**Figure 6:**
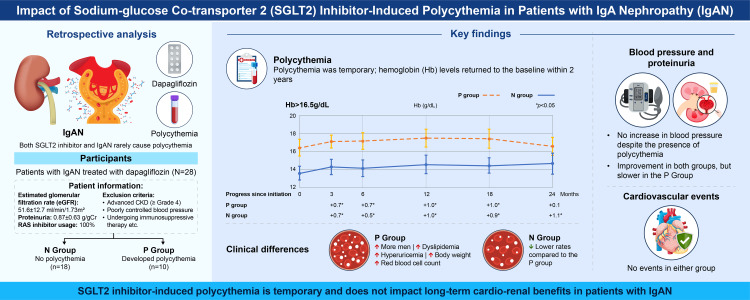
Impact of SGLT2 inhibitor-induced polycythemia in patients with IgAN SGLT2: Sodium-glucose co-transporter 2; IgAN: IgA nephropathy This figure was created by the authors and has not been previously published elsewhere.

## Appendices

Appendix A 

The SGLT2 inhibitor-induced polycythemia is temporary and does not impact long-term cardio-renal benefits in patients with IgAN (Figure 6).
